# Public awareness of audiology, hearing and hearing health in the Limpopo Province, South Africa

**DOI:** 10.4102/sajcd.v64i1.557

**Published:** 2017-09-28

**Authors:** Karin Joubert, Ben Sebothoma, Khomotjo S. Kgare

**Affiliations:** 1 Department of Speech Pathology and Audiology, University of the Witwatersrand, South Africa; 2 Ndlovu Wits Audiology Clinic and Outreach Programme, South Africa

## Abstract

**Background:**

The burden of hearing loss is on the increase, especially in low-income countries such as South Africa. The need for urgent action to prevent ear and hearing problems is a priority, especially as in many cases permanent hearing loss is preventable. In South Africa, as in other developing countries, there is a limited number of hearing health professionals and audiological resources. The lack of hearing health services may impact the general public’s awareness of hearing and hearing health. Limited information is available on the South African public’s knowledge of audiologists and the services they provide, especially in underserved rural communities.

**Aim:**

The aim of this study was to describe individuals’ awareness of the audiology profession, hearing and hearing loss, and hearing health in a rural area of the Limpopo Province.

**Method:**

A cross-sectional survey design was employed for the purpose of this study. Using a random sampling strategy, 297 households in four rural villages were selected and a self-developed questionnaire was administered to one individual (18 years and older) per household. The questionnaire consisted of 23 questions targeting awareness of the audiology profession, as well as knowledge of hearing, hearing loss and hearing health.

**Results:**

Only 14% of participants were aware of the audiology profession, indicating that individuals living in rural communities are not aware of the role of audiologists and the services they provide. Doctors and nurses were identified by participants as the individuals who assist them with hearing-related problems. Although most participants (87%) acknowledged that it is very important to undergo a hearing test, only 5% have previously visited an audiologist. Most participants were aware that ear infections and excessive noise exposure can cause hearing loss. The majority also believed that ears must be kept clean at all times and used cotton buds to maintain ear hygiene.

**Conclusion:**

There is a general lack of public awareness of audiologists and the services they offer. This study highlighted the need for the National Department of Health in collaboration with professional associations and hearing health professionals to develop and implement effective strategies to increase the South African public’s awareness of the profession and the services they provide. South African universities can also play a significant role in teaching students to develop context-relevant strategies to increase awareness of the profession.

## Introduction

Audiologists are health care professionals who assess, diagnose and treat hearing- and balance-related problems (Martin & Clark, 2015). In addition to the clinical aspect of audiology, prevention of hearing impairment and the promotion of hearing health are also critical and therefore incorporated into the realm of audiology (Anderson & Shames, 2011).

Despite the existence of the audiology profession since the 1940s, hearing impairment remains a major public health concern. The World Health Organization (WHO) recently highlighted the importance of intensifying action to prevent deafness and hearing loss as worldwide approximately 360 million people live with disabling hearing impairment^1^ (WHO, 2017). The prevalence of disabling hearing impairment for both adults and children is the highest in South Asia, Asia-Pacific and sub-Saharan Africa (WHO, 2012). In South Africa, the accelerated prevalence of communicable diseases [e.g. HIV and tuberculosis (TB)] and the subsequent use of ototoxic medications have exacerbated the prevalence and nature of hearing loss (Harries et al., 2012).

Information on the prevalence and causes of hearing impairment (across the lifespan) is available for the South African population with the bulk of the research conducted in urban areas and/or within the private health care sector (Meyer & Swanepoel, 2011; Ramma & Sebothoma, 2016; Strauss, Swanepoel, Becker, Eloff & Hall, 2012; Swanepoel, Ebrahim, Joseph & Friedland, 2007; Tiedt et al., 2013).

There is, however, limited information on the prevalence and causes of hearing impairment in rural South Africa. Rural areas in South Africa are defined as remote areas with poor infrastructure, poor basic utility service provision, low levels of literacy, high levels of unemployment, limited access to health and education services and a high incidence of communicable diseases (Watermeyer & Barratt, 2013). Impacted cerumen, middle ear pathology, exposure to excessively loud music and the use of ototoxic medication have been reported as the most prevalent causes of hearing impairment in rural areas (Mulwafu, Kuper & Ensink, 2016; Pullen, 2015; Ramma & Sebothoma, 2016). Most of these causative factors can, however, be prevented, at least partially, by the improvement in the primary prevention of hearing impairment (Olusanya, Neumann & Saunders, 2014).

Strategies for the primary prevention of hearing impairment across the lifespan have been outlined by Olusanya et al. (2014). The guidelines on health education regarding ototoxicity, middle ear pathology, exposure to excessive and/or prolonged noise, the importance of immunisation as well as the promotion of appropriate personal hygiene provide a useful starting point for audiologists and other health care professionals. The implementation of these strategies as well as the early intervention of hearing loss can limit the devastating consequences hearing impairment can have on communication, psychosocial well-being, economic independence and overall quality of life.

There is limited information on the general public’s awareness of audiology and the role of audiologists. An early study on the awareness of college students’ knowledge and awareness of hearing and hearing loss indicate that there is a lack of knowledge and understanding of the existence of and the role of audiologist (Lass, Woodford & Everly-Myers, 1990). Although some survey-based international studies have attempted to provide insight into the general public’s awareness of the audiology, hearing and hearing health (Di Berardino et al., 2013; Gabriel, Mohammed & Paul, 2015; Lass et al., 1990; Lee, Govindara & Hon, 2005; Narayansamy, Ramkumar & Nagarajan, 2014), no information is available for the rural South African context.

## Methodology

### Aim

The aim of this study was to describe individuals’ awareness of the audiology profession, hearing and hearing loss, and hearing health in the rural areas of the Limpopo Province.

### Research context

The research was conducted in four villages in the Elias Motsoaledi Local Municipality (EMLM) area of the Sekhukhune District in the southern part of the Limpopo Province. The Sekhukhune District is one of the poorest districts in the country and is characterised by poor infrastructure and lack of safe water supply (Health Systems Trust, 2017). The EMLM has 62 settlements that are mostly rural villages and has an estimated population of 249 363. Almost 99% of the population are black South Africans. Education levels are low as 46% of the population have no formal schooling. Eleven per cent has completed some form of primary schooling and only 4% of the population have some form of higher education (EMLM, 2013/2014).

### Participants

A systematic random sampling strategy was used to select participants in four villages in the EMLM (Table 1). These villages included Elandsdoorn, Ntwane, Tambo and Phooko. Every third household in each of the villages was visited by the research team and only one adult individual per household was requested to participate in the study. Individuals were only included if written consent was provided.

**TABLE 1:** Number of households surveyed.

Variable	Elandsdoorn	Ntwane	Tambo	Phooko
Number of households surveyed	96	107	25	69

A total of 297 individuals were included in the study. The average age of the participants was 38.5 years (range: 18–69; *±*15.43) and the majority (73%; *n* = 216) were females. The home language of most participants was isiZulu (58%; *n* = 171), followed by Sepedi (21%; *n* = 63), whilst the remaining 21% (*n* = 63) speak a combination of Xitsonga, Tshivenda, isiNdebele and Shona. Most participants (57%; *n* = 169) were unemployed. Only 51% (*n* = 151) of the participants had some form of high school education, and 12% (*n* = 36) had no formal education.

### Measure

A self-developed questionnaire was used to collect data (Appendix 1). The questionnaire consisted of five sections and a total of 23 open- and/or closed-ended questions. The five sections of the questionnaire were demographic information, knowledge and awareness of the audiology profession, hearing and hearing loss, and ear hygiene. The survey questionnaires were administered by trained research assistants fluent in isiZulu, Sepedi, Xitsonga, Tshivenda, isiNdebele and English.

A pilot study was conducted prior to data collection. The research team comprised an audiologist and six research assistants (community members specifically trained for the purpose of the study). Each research assistant was required to administer the questionnaire on five community members who met the same inclusion criteria as for the main study. The 30 pilot study participants resided in the same communities where the research was conducted. The objectives of the pilot study were to ensure the face and content validity of the questionnaire as well as to ensure that there were no discrepancies with regard to the documentation of participant responses.

### Procedure

#### Ethical considerations

Ethical clearance was obtained from the Human Research Ethics Committee (Medical) of the University of Witwatersrand before the study commenced (Protocol number M121006). Permission to conduct the study was obtained from the relevant tribal authorities (e.g. chiefs). Every third household in each of the villages was visited by the research team. Individuals in each of the selected households were informed of the study and only one individual per household was requested to participate in the study. Participants were fully informed about the nature of the study, and assured of confidentiality and their rights to withdraw from the study at any time without negative consequences. Only participants who gave written consent were included in the study. Participants who required hearing health care were referred to their local primary health care clinic or the audiology department. All completed questionnaires were verified for completeness by the audiologist and data were captured on a password-protected Excel spreadsheet. Raw data were stored in a locked cabinet and will be destroyed after a period of 5 years.

## Results

The results will be presented as it relates to the: (1) awareness of the audiology profession, (2) hearing and hearing loss and (3) hearing health.

### Awareness of the audiology profession

The majority of participants (86%; *n* = 255) were not aware of the audiology profession. A large majority indicated that other health professionals can test hearing (Table 2).

**TABLE 2:** Assistance with hearing problems (*n* = 297).

Variable	*n*	%
**Who can test your hearing?**		
Medical doctor	218	73
Audiologist	42	14
Clinic nurses	24	9
I don’t know	13	4
**Where can your hearing be tested?**		
Clinic	167	56
Hospital	48	16
Private doctor	44	15
I don’t know	34	13

Only 14% (*n* = 42) of the participants were aware that audiologists are the health professionals responsible for testing hearing (*n* = 15) and to help with ear problems (*n* = 22). These participants indicated word-of-mouth (21%; *n* = 8) and information from other health workers (30%; *n* = 11) as their sources of information.

### Awareness of the hearing and hearing loss

Despite the lack of awareness about the audiology profession, many of the participants (87%; *n* = 257) agreed that it was ‘greatly important’ to have their hearing tested. Only two participants indicated that it was ‘not important at all’. The majority of participants (92%; *n* = 273) agreed that everyone’s hearing can be tested regardless of age. Interestingly, only 5% (*n* = 16) of participants have previously visited an audiologist for a hearing test.

Participants were asked, using an open-ended question, what can be done if they have a hearing problem. A variety of responses were reported (Table 3).

**TABLE 3:** What can be done if you have a hearing problem (*n* = 297).

Variable	*N*	%
Consult a doctor	89	30
Get treatment	67	22
Consult at the clinic	45	15
I don’t know	30	10
Get a hearing aid	20	7
Consult the audiologist	14	5
Consult a specialist	12	4
Undergo an operation	8	3
Consult at the hospital	7	2
Other (e.g. counselling, nothing, go to church)	5	2

### Awareness of hearing health

Participants were questioned regarding the awareness of ear infection, noise exposure and ear hygiene.

#### Ear infection

When asked whether an ear infection can cause hearing loss, the majority of the participants (87%; *n* = 257) indicated that ear infections can cause hearing loss. Most participants (65%; *n* = 193) specified that they ‘hardly ever’ have ear infections, whilst only 5% (*n* = 15) have ear infections ‘frequently’ and 3% (*n* = 9) ‘almost always’. Interesting responses were recorded regarding the actions that participants take when they have an ear infection. The majority 38% (*n* = 114) indicated that they consult with medical professionals at a clinic or hospital, whilst 7% (*n* = 22) reported that they do nothing. Twelve per cent (*n* = 37) reported that they insert oil in their ears. The types of oil inserted included sweet oil (*n* = 12), cooking oil (*n* = 9), fish oil (*n* = 6), chicken fat or oil (*n* = 5), glycerine (*n* = 4) and castor oil (*n* = 1). Only one participant reported consulting a traditional healer for treatment of hearing loss or/and ear infection.

#### Excessive noise exposure

Excessive noise exposure was also probed. An overwhelming majority (89%; *n* = 265) agreed that excessively loud noise can damage hearing. Participants were required to indicate which type of noise typically found in their village can cause damage (Table 4). Listening to loud music in a taxi or on an MP3 player was reported to mostly cause damage.

**TABLE 4:** Type of excessive noise that can damage hearing (*n* = 297).

Type of excessive noise	*n*	%
Music in taxi	89	30
Listening to MP3 player	86	29
Listening to cell phone	49	16
Music in church	4	1
All of the above	45	15
None of the above	23	9

The last question probed how participants thought they can protect their hearing from excessive noise. The results are presented in Table 5. The majority of participants indicated that they can protect their ears by avoiding loud music (38%; *n* = 112), lowering the volume of the music they are listening to (19%; *n* = 55) and using some form of ear protection, such as cotton wool and ear plugs (18%; *n* = 55).

**TABLE 5:** Ways to protect hearing from excessive noise (*n* = 297).

Ways to protect hearing	*N*	%
Avoid loud music or sounds	112	38
I don’t know	59	20
Lower the volume of music	55	19
Use earplugs	37	13
Put cotton wool in your ears	14	5
Nothing can protect your ears	8	3
Ask the doctor or audiologist	5	1.7
Clean ears regularly	3	1
Do not listen to music through earphones	3	1
Only God can protect your ears	1	0.3

#### Ear hygiene

**FIGURE 1:**
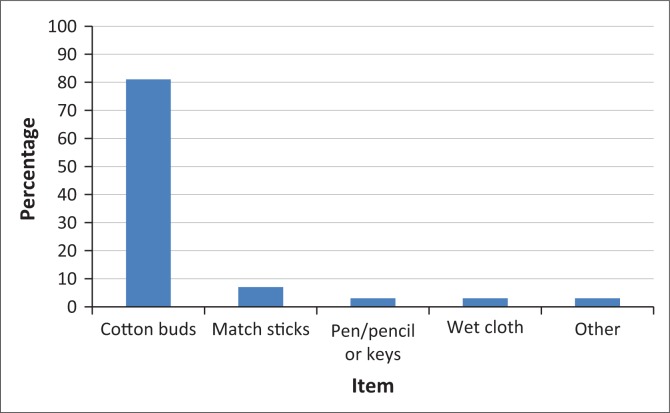
Items used for ear hygiene.

Most of the participants believed that ears must be kept clean at all times and indicated that they use cotton buds, matchsticks and a variety of other items to clean their ears (Figure 1). Of the participants who indicated that they use cotton buds to clean their ears, 64% (*n* = 190) also use it when their ears are itchy.

## Discussion

There is a general lack of awareness of audiology as a profession in the community where the research was conducted. This is despite the availability of audiological services at the two hospitals and one local non-government clinic in the EMLM area. It is postulated that health care workers (professional nurses and doctors) based at the two hospitals and various primary health care clinics are not aware of the availability of audiological services in the EMLM. Participants who were aware of the audiology profession indicated that they were made aware of the services offered primarily by other health care workers and also through word-of-mouth. This supports the notion that the primary source of health-related information is through health workers who do health talks in hospitals and clinics (Gabriel et al., 2015).

Only 10% (*n* = 30) of the participants did not know what to do if a hearing problem is noted. The majority of participants (78%; *n* = 234) correctly indicated that they will consult a health care worker (e.g. nurse, doctor and specialist) at either the clinic or the hospital. Ten per cent (*n* = 28) also mentioned treatment options such as being fitted with a hearing aid or undergoing an operation. The consultation sources and suggested treatment options mentioned by participants are appropriate. However, without referral by health professionals to audiologists, the early identification and appropriate management of hearing impairment (e.g. hearing amplification devices and aural rehabilitation) may impact the quality of life of individuals with hearing impairment (Olusanya et al., 2014).

The participants’ awareness of ear infection as a cause of hearing impairment was good. The action that they would take if they have an ear infection ranged from doing nothing, consulting with health professionals at the clinic or hospital to treating it themselves by inserting a substance (usually a type of oil) into the ear. The practice of inserting oil to treat ear infection has also been reported in rural African communities (e.g. Central Kenya) (Njoroge & Bussmann, 2006). In Kenya chicken fat, other industrial lubricants and 13 plant species were used to manage ear infection.

Participants were further aware of the impact of excessive and prolonged music exposure on hearing. The most prevalent form of noise exposure in these rural villages is music. Music is often played very loudly in the close confines of taxis and shebeens as well as at most events and even in churches. The use of cell phones and MP3 players is also quite high as most teenagers and young adults use earphones to listen to music with these devices. The strategies suggested by the participants to protect their hearing from excessive noise exposure were mostly appropriate (e.g. avoid loud music, lower the volume and use ear plugs). However, some participants offered inappropriate suggestions (e.g. use cotton wool, clean ears regularly) or did not know how to protect hearing from excessive noise.

Ear hygiene practices comprised mostly the use of objects (cotton buds, matchsticks and pen or pencil) to clean or scratch ears. This is despite the potential complications associated with the use of these objects in ears. Common complications associated with the use of cotton buds include otitis externa, otomycosis, laceration in the external auditory meatus, accumulation of wax and perforated tympanic membrane (Lee et al., 2005). Similar ear cleaning practices were reported in other studies conducted in developing countries such as Malaysia, Nigeria and South India (Gabriel et al., 2015; Lee at al., 2005; Narayansamy et al., 2014).

## Conclusion

The findings of this study highlight the urgent call for action by all relevant stakeholders to increase existing public awareness of the audiology profession, hearing and hearing health services especially in the rural areas of South Africa.

### Recommendations

Based on the findings of the current study, it is recommended that health education for this rural population should focus on the following: (1) the audiology profession and its role in the identification and management of hearing- and balance-related problems, (2) the availability of audiological services in the area, (3) the impact of excessive noise exposure on hearing and strategies on how to protect hearing and (4) ear hygiene, specifically why the use of cotton buds is not advised.

It is, however, imperative that the National Department of Health prepare, implement and monitor a national plan for the prevention and control of major causes of avoidable hearing impairment within the framework of primary health care (Olusanya et al., 2014). Collaboration with professional hearing health organisations or associations, non-governmental organisations and hearing health professionals is required to support and coordinate these programmes. These programmes should not only be confined to health care facilities in urban areas, but should also be expanded to under-resourced rural areas. An important component of such a programme should be the development of culturally and contextually appropriate information and education for hearing protection and conservation for both the public and health care workers. It is essential that health care workers, especially doctors and nursing staff, at all levels of care should be educated about the role of the audiologist in the identification and management of hearing-related problems.

It is further recommended that similar studies should be conducted in a variety of other contexts to determine the extent of education required to prevent avoidable hearing- and balance-related problems.

## Footnotes

1 Disabling hearing impairment: hearing loss > 40 dB in the better hearing ear in adults and > 30 dB in children.

## Appendix 1

**Questionnaire**

Participant no: ________________________________

Research assistant no: __________________________

Date of completion: ____________________________

**Instructions for completion:** Please answer all the questions

**Demographic information**

**Table:**

Date of birth: ____________________________	Gender: Male or Female
Highest level of education: _________________	Occupation: __________________________
Home language: _________________________	Area: ________________________________
Closest clinic: ___________________________	Closest hospital: _______________________

**Knowledge of the profession**

1. What does an audiologist do?
2. Where is your closest audiologist? (only if the participant knew what an audiologist is)
3. Have you ever visited an audiologist? No or Yes If yes, why?
4. From which source did you find out about an audiologist?
  - Word-of-mouth
  - Health workers
  - Radio
  - TV
  - Other (*please specify*)

**Knowledge or awareness of hearing and hearing loss**

5 How important is it to have your hearing tested?
  - Greatly important
  - Considerably important
  - Important
  - Somewhat important
  - Not important at all
6 Whose hearing can be tested?
  - Babies
  - Children
  - Teenagers
  - Young adults
  - Adults
  - Everyone
7 Who can test your hearing?

**Hearing**

8 What do you think is the cause of hearing difficulties?
  - Ear infection
  - Noise (e.g. MP3 players, music)
  - Some medications (e.g. TB, HIV, Malaria, etc.)
  - Family member having a hearing loss
  - Wax
  - None of the above
  - Other
9 How would you know if you have a hearing loss?
10 Where do you go for help when you have a hearing problem?
  - Clinic
  - Traditional healer
  - Church
  - Doctor
  - No one
  - Other (specify)
11 Where can your hearing be tested?
  - Clinic
  - Hospital
  - Private Doctor
  - I do not know
  - Other (specify)
12 What can be done if you have a hearing problem?

**Ear infections**

13 Can an ear infection cause a hearing loss?
  - Yes
  - No
  - Maybe
  - I do not know
14 How often do you have ear infections?
  - Hardly ever
  - Occasionally
  - Sometimes
  - Frequently
  - Almost always
15 What do you do when your ears are painful?
16 What do you do if you have an ear infection?

**Ear hygiene**

17 How often do you clean your ears?
  - Hardly ever
  - Occasionally
  - Sometimes
  - Frequently
  - Almost always
18 What do you use to clean your ears?
  - Cotton buds (ear buds)
  - Matchsticks
  - Pen or pencil
  - Wet cloth
  - nothing
  - Other (*please specify*)
19 What do you use when your ears are itchy?
  - Cotton buds (ear buds)
  - Matchsticks
  - Pen or pencil
  - Finger
  - Nothing
  - Other (*please specify*)

**Noise versus hearing**

20 Do you think music or noise can damage your hearing?
  - Yes
  - No
  - Maybe
  - I do not know
21 Do you think excessive loud noise can damage your hearings?
  - Yes
  - No
  - Maybe
  - I do not know
22 Which of the following do you think can damage your hearings?
  - Music in taxi
  - Music in church
  - Listening to MP3 player
  - Listening to cell phone
  - All of the above
  - None of the above
23 How would you protect your hearing from being damaged by excessive loud noise?
